# Treatment Technology of Hazardous Water Contaminated with Radioisotopes with Paper Sludge Ash-Based Geopolymer—Stabilization of Immobilization of Strontium and Cesium by Mixing Seawater

**DOI:** 10.3390/ma11091521

**Published:** 2018-08-24

**Authors:** Zhuguo Li, Mariko Nagashima, Ko Ikeda

**Affiliations:** 1 Department of Architectural Design and Engineering, Graduate School of Science and Technology for Innovation, Yamaguchi University, Ube 755-8611, Japan; li@yamaguchi-u.ac.jp; 2 Department of Earth Sciences, Graduate School of Science and Technology for Innovation, Yamaguchi University, Yamaguchi 753-8512, Japan; nagashim@yamaguchi-u.ac.jp

**Keywords:** geopolymer, paper sludge ash, radionuclide, hazardous water, immobilization, seawater, strontium, cesium, chlorine

## Abstract

Long-term immobilization ratios of strontium (Sr^2+^) and cesium (Cs^+^) in paper sludge ash-based geopolymer (PS-GP) were investigated in one year. PS-GP paste specimens were prepared in the conditions of 20 °C and 100% R.H., using two kinds of paper sludge ash (PS-ash). Two kinds of alkaline solution were used in the PS-GP as activator. One was prepared by diluting aqueous Na-disilicate (water glass) with seawater. Another was a mixture of this solution and caustic soda of 10 M concentration. When seawater was mixed into the alkaline solution, unstable fixations of Sr^2+^ and Cs^+^ were greatly improved, resulting stable and high immobilization ratios at any age up to one year, no matter what kind of PS-ash and alkaline solution were used. Element maps obtained by EPMA exhibited nearly even distribution of Cs^+^. However Sr^2+^ was biased, making domains so firmly related to Ca^2+^ presence. The mechanism that seawater stabilizes immobilization of Sr^2+^ and Cs^+^ was discussed in this study, but still needs to further investigation. Chemical composition analyses of PS-GP were also conducted by SEM-EDS. Two categories of GP matrix were clearly observed, so called N-A-S-H and C-A-S-H gels, respectively. By plotting in ternary diagrams of SiO_2_-(CaO + Na_2_O)-Al_2_O_3_ and Al_2_O_3_-CaO-Na_2_O, compositional trends were discussed in view of ‘plagioclase gels’ newly found in this study. As a result, it is suggested that the N-A-S-H and C-A-S-H gels should be strictly called Na-rich N-C-A-S-H and Ca-rich N-C-A-S-H gels, respectively.

## 1. Introduction

Conventionally, by using minerals as immobilization media, various attempts have been made so far to remove hazardous elements from water contaminated with heavy metals. Representative minerals are zeolite, apatite, etc. Among others, tobermorite is peculiar [1]. Cementitious materials are generally used to treat contaminated sludge with less water. However, Portland cement paste or concrete has incomplete immobilization capabilities, especially for lead and sometimes zinc [2,3]. On the other hand, geopolymer as other kind of immobilization media has attracted attention in recent years [3,4,5,6,7,8,9,10,11]. The immobilization principle of geopolymer is the polycondensation of silicic acid monomers, in which the foreign ions are incorporated into the siloxane bonds of tetrahedra to promote polymerization of monomer [SiO_4_]-complex associated with other supplemental coordination sites to maintain charge neutrality. Therefore, it is possible to fix toxic metals into geopolymer. However, geopolymer is versatile, it is unsuitable for immobilization of arsenic [9]. The present situation of immobilization of toxic elements using geopolymer are reviewed recently in the literature [12]. 

Radionuclide-contaminated waste is classified into high dose and low dose pollutant waters. In the former, it is vitrified by melting to place into containers called canister and are permanently stored in deep underground, which is called geological disposal. In the latter, it is solidified by pitch or cement, then put into drums, and finally discarded underground, which is called shallow burial. The urgent problem is an unexpected leakage of radionuclides, happening in the nuclear reactors of Fukushima Daiichi Nuclear Power Plant due to meltdown caused by the large earthquake and subsequent tsunami. The leaked radionuclides have been spreading out in contaminated water due to infiltration of groundwater to cause serious environmental problem. Even though the radionuclides’ doses are not so extremely high, it is a tedious work to treat a huge amount of the contaminated water. Current treatment is in a two-stage plant equipped with SARRY and ALPS. In the first stage cesium is adsorbed with zeolite and Ti-silicate. In the second stage, the rest 62 radionuclides are eliminated in addition to cesium uncaptured and leaked from the first stage by a flowline installed iron coprecipitation, carbonate coprecipitation, titanium oxide adsorbent columns, etc. However, since radioactive tritium cannot be removed, the treated water has been still stored in several hundred tanks. The storage tanks continue to increase day by day. Furthermore, in the long term, there is a concern about leakage from the water tanks due to the metal tanks corroding. Therefore, it is urgently necessary to treat the contaminated water rapidly in bulk quantities. Incidentally, though tritium is radioactive, it is said that tritium does not cause serious health problems, since ingested tritium would be discharged together with urine promptly. Therefore, it is theoretically no problem to run out the treated water into open sea. However, fishermen around the Fukushima Daiichi Nuclear Power Plant are fiercely opposed out of fear of a rumor that the water would poison fish, so the treated water has been kept in the storage tanks.

For this reason, the authors developed the effective method as described in the previous reports to solve this problem [4,5]. That is, papermaking sludge incineration ash (PS-ash) is mixed with the alkaline solution to make geopolymer. The alkaline solution is prepared by adding the contaminated water. Since PS-ash is porous, it can absorb a very large amount of liquid, when using it as active filler or precursor of GP. It has been confirmed that the PS-ash-based geopolymer (PS-GP) may treat hazardous water contaminated with radionuclides. One ton of PS-ash can treat more than one ton of contaminated water, and the immobilization ratios of Sr^2+^ and Cs^+^ are generally very high, showing more than 90%. However, some serious issues have been encountered after the previous works [4,5] as well as after continuous measurement up to one year later. That is, the immobilization ratios of Sr^2+^ and Cs^+^ in the PS-GP added with non-radioactive strontium nitrate and cesium nitrate as surrogates are unstable and fluctuating.

Now the damaged nuclear reactors are cooled by fresh water. However, in the beginning of the Fukushima Daiichi Nuclear power plant disaster, seawater was actually pumped up from the port in front of the premises to cool down the damaged nuclear reactors as an emergency measure. Hence, in this context, we used seawater to prepare alkali silicate solution to produce PS-GP, and found that the immobilization ratios of Sr^2+^ and Cs^+^ became stable even in long-term. In this study, we aim to clarify the long-term immobilization ratios of Sr^2+^ and Cs^+^ in PS-GP and the immobilization mechanism in view of polycondensation products of PS-GP, when the alkali silicate solution is prepared by adding sea water.

## 2. Materials and Methods 

### 2.1. Preparation of Alkali Silicate Solutions

Commercially available water glass, called JIS (Japanese Industrial Standards) No. 1 sodium disilicate aqueous solution (nominal composition, Na_2_O·2SiO_2_·aq), was diluted with deionized water to prepare a sodium silicate stock solution with specific gravity (S.G.) of 1.54. Then, seawater was added to the stock solution to obtain the first alkali silicate solution with S.G. 1.27, which is called GP-liquor #1SW. Furthermore, the GP-liquor #1SW was mixed with caustic soda aqueous solution of 10 mole concentration by a volume ratio of 3:1 to obtain another solution with S.G. 1.30, which is called GP-liquor #0SW. Details are shown in Table 1. 

The used seawater was retrieved from the outside of the Yakeno Coast breakwater of the Seto Inland Sea, Sanyo-Onoda City, Yamaguchi Prefecture, Japan, at the time of high tide. Prior to using, the seawater was percolated by filter paper of no. 131. The general S.G. of Pacific seawater is in the range of 1.02–1.03, but the Seto Inland Sea is slightly heavy, as measured 1.04 of S.G.

### 2.2. Preparation of Specimens and Strength Test

Chemical compositions of two kinds of PS-ash used as active fillers of PS-GP are shown in Table 2 together with their physical properties, which were determined by conventional techniques, including X-ray fluorescent analysis (XRF, MagixPro, Royal Philips, Amsterdam, Holland), and Blaine specific surface area measurement (Marubishi Kagaku, Tokyo, Japan). The PS-ash, called OTo3, is characterized by high content of Al_2_O_3_ component, whereas N45 has high CaO and MgO components. Details are kindly referred to the previous works in addition to constituent minerals of the PS-ashes [4,5].

PS-GP bodies were respectively prepared from the two kinds of PS-ash. As shown in Table 3, the liquor/filler ratio (L/F) varied with the type of PS-ash. 100 grams of the PS-ash sample was weighed, and the GP-liquor was mixed to the limit amount, at which bleeding did not occur. Then, the mixture was hand-mixed in a 500 mL plastic beaker and cast into a metallic mold consisting of three prismatic cells. Each cell has 20 × 20 × 80 mm dimension. Grease was preliminarily smeared to the interior of the mold for easily demolding. In order to reduce the dry shrinkage of PS-GP before demolding, the GP specimens were placed in the sealed plastic chamber that was 20 °C and had shallow water in its bottom so that it had nearly 100% R.H. After being cured in the humid air for 24 h, the GP specimens were demolded, the curing was continued in the same conditions until 28 days age. The bulk densities of the PS-GP bodies were measured right after the 28 days curing on the basis of mold volume, and the three-point bending test was conducted. The span of specimen in the bending test was 50 mm and the loading speed was 0.2 mm/min. Either flexural strength and bulk density was an average of three specimens. Extra PS-GP bodies were continued to cure in the ambient air of 20 ± 3 °C. Then, at the ages of 6 (4 + 2), 12, 24, and 52 weeks (≈1 year), bulk densities were measured again to determine the strontium and cesium contents of the PS-GP samples.

### 2.3. Dissolution Test

The water discharged from the Fukushima Daiichi Nuclear Power Plant is contaminated mainly by ^90^Sr and ^137^Cs in addition to ^134^Cs. Specifically, the former two radionuclides have very long half-lives of nearly 30 years. In this study, nonradioactive nitrate reagents (Sr(NO_3_)_2_, CsNO_3_) were added as surrogates at a ratio of 1% by mass, respectively, to the PS-ash. As the dosages of nitrate reagents were very small, they were excluded from the calculation of liquor/filler ratio (L/F) for convenience. The radiological dosage of contaminated water of Fukushima Daiichi nuclear power plant is estimated to be 10^8^ to 10^9^ Bq/L. In contrast, the surrogate nitrates added to the PS-GP correspond to a level of 10^12^ Bq/L, thus the addition of nitrates was sufficient. The same addition of nitrates was also done to the PS-GP without mixing seawater in previous works, in which the dissolution test of Sr^2+^ and Cs^+^ was conducted only up to 24 weeks [4,5]. The previous test results are shown again in this paper, together with the data newly obtained in this study at 52 weeks.

The dissolution test method is as follows: the PS-GP body was firstly pulverized to the size under 4 mm. Then, the 12.5 g sample was filled into a 250 mL wide-mouth plastic bottle and the acid water was also poured into a bottle. The water mass was 10 times that of the GP sample. Hydrochloric acid or glacial acetic acid is generally used as leaching fluid, well-known as JLT-13 (pH 6.3) and TCLP (pH 2.88) respectively, but for the convenience of our laboratory facility, a standard buffer solution of phthalate salt of pH 4.01 was used in this study [4]. The bottle was cap-sealed and rotated for 6 h under a rotating speed of 60 rpm. The mixture in the bottle was then filtered with a qualitative filter paper no. 131, and the filtered leachate was further diluted up to 10 times by volume with pure water to use as the sample of the dissolution test. Finally, the dissolution concentrations of Sr^2+^ and Cs^+^ were measured by induction coupled plasma atomic emission spectrometry (ICP-AES, Optima 8300, PerkinElmer, Waltham, MA, USA).

The dissolution ratio was calculated as following procedure: Firstly, comparing the bulk densities at each age with the four weeks to determine the PS-ash amount used to produce the 12.5 g GP sample, the decrease of density of the test sample is simply due to evaporation of constituent water. Then, the amounts of the surrogates included into the 12.5 g GP sample were determined. Secondly, dissolution ratios were obtained on the basis of the ICP results and the charged fluid amount that is 125 grams. Finally, the immobilization ratio was obtained simply as

% immobilization ratio = 100 − % dissolution ratio(1)

More details can be kindly referred to in the previous works [4,5].

### 2.4. XRD Analysis

In order to clarify the phases of PS-GP body, XRD analysis was carried out for the specimens with one-year age, employing RIGAKU RINT-2250 (Rigaku, Tokyo, Japan). Measuring conditions were 40 kV−200 mA monochromatic CuKα radiation, 1°−1°−0.3 mm slit system, 0.02 degree step scan, and 4°/min scanning speed.

### 2.5. EPMA Analysis

Element mapping analysis was carried out for the one-year old specimens, employing an electron probe micro analyzer (EPMA, JEOL JXA-8230, Jeol, Tokyo, Japan). Measuring conditions were 15 kV acceleration voltage and 20 nA irradiation current. Peak intensity positions of the elements analyzed in this study were determined by using the standard samples in advance. The PS-GP sample was gathered by cutting the PS-GP body with a 0.5 mm thick diamond blade, thoroughly dried after washed with ethanol, and then subjected to impregnation by embedding with epoxy resin. After preparing a thin slice, which was stuck to a slide glass as conventionally done in petrology discipline, then mirror finish was applied with diamond paste and finally subjected to vapor deposition with carbon.

## 3. Results and Discussion

In the present study, generally accepted terminologies of ‘N-A-S-H’ and ‘C-A-S-H’ are still used to describe the resultant matrix gels for convenience [13,14,15], though strictly speaking, they should be called ‘Na-rich N-C-A-S-H’ and ‘Ca-rich N-C-A-S-H’ gels, respectively, as concluded later. Moreover, the term of ‘GP-minerals’ is used to express crystalline phases presented finally in hardened GP bodies except amorphous N-A-S-H and C-A-S-H gels, where C, N, A, and H denote CaO, Na_2_O, Al_2_O_3_, SiO_2_, and H_2_O, respectively.

### 3.1. Strength and Density of PS-GP Body

As shown in Table 3, the PS-GP using the seawater had a larger liquor/filler ratio (L/F), compared to the series without mixing the seawater. That is to say, the addition of seawater makes the PS-GP able to uptake much contaminated water. Just because the PS-GP using the seawater-containing GP-liquor #0SW had a higher L/F, its flexural strength was smaller than the counterparts of non-seawater mixing PS-GP. Series 1-1-SCSW, using the GP-liquor #1SW and the PS-ash OTo3, exceptionally shows a higher flexural strength. The reason is unknown at this moment.

In the case of adding the seawater, the bulk density of PS-GP was not significantly different from the non-seawater mixing PS-GP at all the age, despite the L/F of the former was larger. However, the bulk density of the series 0-1-SCSW, using the GP-liquor #0SW and the PS-ash OTo3, exceptionally had a lower bulk density. Remarkable foaming and more porous features were observed in 0-1-SCSW specimens, resulting in lower bulk density. Thus, water evaporation easily occurred through continuous pores, which were confirmed by the floating test of specimen in water vessel. In the beginning, the specimen floated, but soon after it sank to the bottom of water vessel. Slight foaming, not to affect the density markedly, was also observed in 1-1-SCSW specimens. It is considered that the foaming is resulted from metallic aluminum included in the PS-ash, which generates hydrogen gas in alkaline GP-liquors. Refuse derived fuel (RDF) is used in some incineration plants. Thereby, aluminum foil appliances, sometimes found in refuse collected from households, may be not completely oxidized during the incineration process and may evaporate to precipitate into the PS-ash as fine particles.

Next, some increase of the bulk density with the elapsed time, found in some seawater-mixed specimens at 24 weeks, should be mentioned. The increase may be firstly caused by damp in rainy season around the 24 weeks. Not only porous characteristics but also hygroscopic nature of chlorine may promote moisture absorption. Secondly, the abnormally high increase of 0-3-SCSW may be caused by the presence of fissures filled with chlorine or chloride which may play an increased role in moisture absorption. The same reason was thought for 1-1-SCSW at 12 weeks when it was not in rainy season. Presumably, the fissures formed between 12 and 24 weeks for 0-3-SWSC and between 6 and 12 weeks for 1-1-SWSC, judging from the sudden increase of the bulk density. The fissures can be kindly referred to Section 3.4.

### 3.2. Dissolution Test of Strontium and Cesium 

Two calculating examples of dissolution and immobilization ratios at 52 weeks are shown in Table 4 for the seawater-mixed PS-GP and the non-seawater-mixed PS-GP specimens, respectively. All the results of dissolution and immobilization ratios of the seawater-mixed PS-GP are summarized in Table 5, in comparison with those of non-seawater-mixed PS-GP. The SrO component was also detected in PS-ashes, but it was not taken into account since the SrO amount was less than a few-hundredth percent orders (see Table 2). 

For the seawater-mixed PS-GP, the immobilization ratios of Sr^2+^ and Cs^+^ were stable within one year, regardless of the types of GP-liquor as well as the types of PS-ash used, and the over-scale (O. S.) was not met, which occurred in some of the non-seawater-mixed PS-GP specimens. However, at 52 weeks, a slight decrease in the immobilization ratios of Sr^2+^ and Cs^+^ were found in the PS-GP, which used the GP-liquor #0SW. As an overall trend, the PS-GPs, which used the GP-liquor #1SW, gave good results of the immobilization ratios of Sr^2+^ and Cs^+^. In addition, the GP-liquor #1SW has an advantage of relatively low cost over the GP-liquor #0SW that contains caustic soda.

On the other hand, the immobilization ratios of Sr^2+^ and Cs^+^ of non-seawater-mixed PS-GP were unstable, fluctuating with the elapsed time. This phenomenon was found especially from series 1-3-SC using PS-ash N45. However, series 1-1-SC using PS-ash OTo3 was an exception and the immobilization ratios were relatively stable within the experimental age. The sort of PS-ash probably is another influencing factor of the immobilization ratios.

However, the most important factor may be the issue that the stability of C-A-S-H and N-A-S-H gels with progress of material age. As elucidated in literatures [16,17], these two kinds of gels are far separated in a ternary diagram SiO_2_-CaO-Al_2_O_3_. With progress of material age, these gels come closer and line up alongside SiO_2_-CaO line, as also found by Yamaguchi et al. [18]. Since the chemical compositions of these gels are instable, the fixation of strontium and cesium may become unstable accordingly. It is strongly estimated that the seawater, most probably chlorine, suppresses the instability of these gels, thus more stable gels may form at earlier age. More detailed discussion will come up in Section 3.4 about this issue.

### 3.3. XRD Results

**Figure 1:**
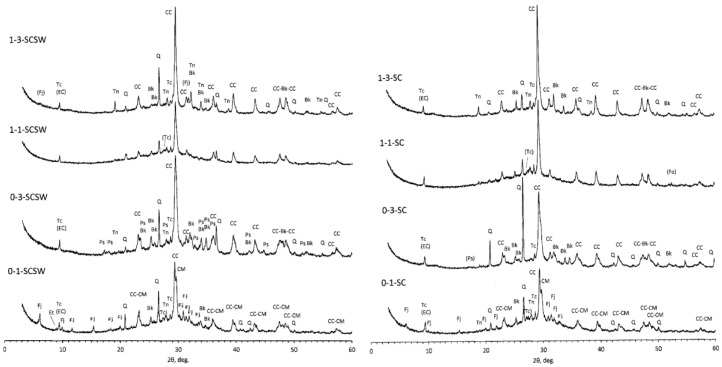
XRD diagrams of seawater-mixed and non-seawater-mixed PS-GP at 52 weeks. CC: calcite; CM: magnesian calcite; Q: quartz; Tc: talc; Fo: forsterite; Fj: faujasite; Bk: burkeite; Tn: thenerdite; Ps: pirssonite; Et: ettringite; EC: carbonate ettringite; ( ): uncertain.

XRD results are represented in Figure 1 for seawater-mixed and non-seawater-mixed PS-GP at the age of 52 weeks. Two categories of PS-GP showed the similar patterns in XRD diagram. Firstly, faujasite formation is peculiar to 0-1-SCSW and 0-1-SC samples as well as presumably in 1-3-SCSW and 1-3-SC samples. Although faujasite peak is unclear in the XRD chart of 1-3-SC at 52 weeks, it was clearly detected at 4 weeks [5]. Therefore, 1-3-SC was instable with elapsed time. Secondly, magnesian calcite was found in both 0-1-SCSW and 0-1-SC samples. Thirdly, pirssonite formed in 0-3-SCSW as well as presumably in 0-3-SC.

Remaining quartz, calcite, and talc were still observed in the PS-GP samples, which are constituent minerals of the raw PS-ashes used in this study, designated as “minerals of primary origin” in our past paper [5]. A few remained ettringite was observed in 0-1-SCSW sample, which is also one of the constituent minerals of the raw PS-ash, designated as “minerals of secondary origin” [5].

In our past studies [4,5], we considered that talc and ettringite completely disappeared from the PS-GPs at four weeks, and carbonate ettringite was produced by the reaction of ettringite and calcite. However, according to the XRD and SEM-EDS results at 52 weeks, as described later in Section 3.5, it was found that part of talc did not react completely and still remained in the PS-GPs. Thus, the formation of carbonate ettringite is implausible. This misunderstanding was caused by overlapping the main peak positions (≈9°, 2θ) between talc and carbonate ettringite. Moreover, forsterite seems to gradually react and will be exhausted with the elapsed time, and its trace was only found in 1-1-SC sample at 52 weeks.

Crystalline GP-minerals, including thenardite, Na_2_SO_4_, and burkeite, Na_6_(CO_3_)(SO_4_)_2_ previously called “minerals in PS-ash based geopolymers” [5], were fundamentally identified in different PS-GP samples, as seen in Figure 1. There was faujasite, (Na_2_, Ca, Mg)_3.5_(Al_7_Si_17_O_48_)·32H_2_O), only in the 0-1 series samples, and its trace may be found in 1–3 series samples. If compared with the XRD charts of 0-1 series samples, seawater addition seems to promote faujasite formation, as seen in the chart of 0-1-SCSW. On the other hand, pirssonite, Na_2_Ca(CO_3_)_2_·2H_2_O, presented only in the 0-3 series samples, especially, it was clearly found in the seawater-mixed PS-GP sample of 0-3-SCSW. As amorphous minerals, N-A-S-H and C-A-S-H gels were observed as a hump in the range approximately 20–40°, 2θ, especially, they were clear in the 0-1 series samples, no matter whether the seawater was mixed or not.

### 3.4. EPMA Analysis of One Year Old Seawater-Mixed PS-GP

#### 3.4.1. Back Scattered Images and Al-Si Distributions

**Figure 2:**
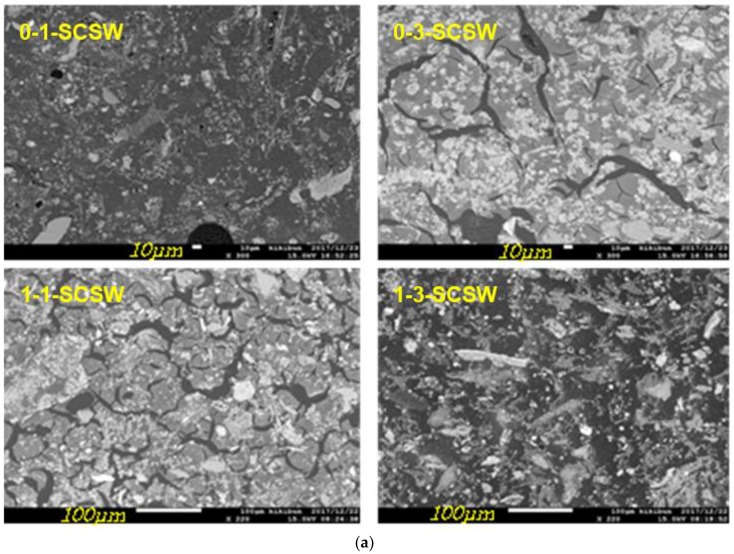

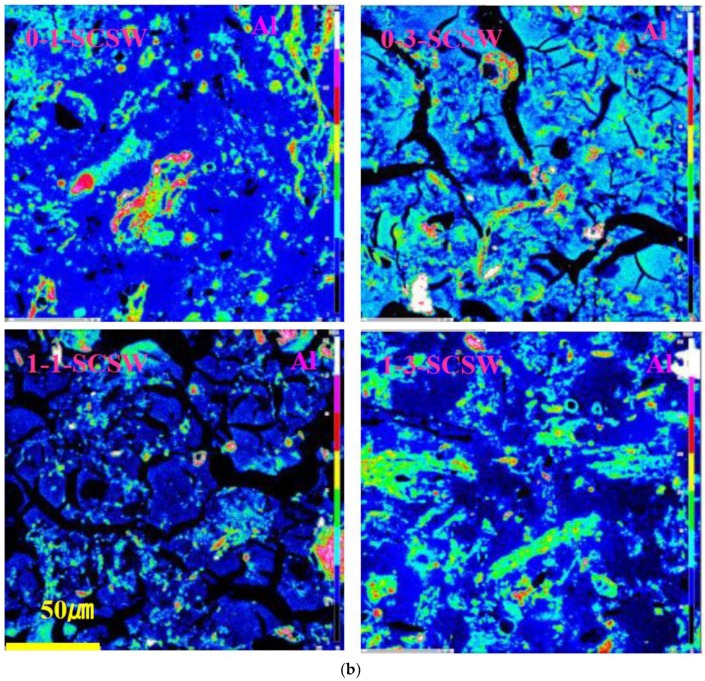
Back scattered electron images (**a**), and Al-distribution maps; (**b**) of PS-GP taken by EPMA. All the scale bars are 50 mm in length for (**b**).

Back scattered electron images of EPMA are shown in Figure 2, together with Al-distribution maps, which well describe GP matrix textures. Large pores resulted from foaming were able to observe with the naked eye in 0-1-SCSW specimen, as studied previously without using seawater [4,5], and small round-shaped voids were also noticed in the back scattered image, which were presumably resulted from air trapping during GP mixing. 1-3-SCSW sample had no visible big pores generated by foaming, but there were small round-shaped voids caused by air trapping too. On the other hand, other samples, 0-3-SCSW and 1-1-SCSW, had crescent lake-like fissures, which are thought to be caused by delayed foaming after setting. However, the destruction of hardened GP bodies, caused by the delayed foaming, did not take place.

From the back scattered electron images, it is very easy to recognize matrix formation. Dark wide areas, looking like sea, are so-called N-A-S-H gels, whereas bright areas, looking like islands, are C-A-S-H gels. It is noted that N-A-S-H and C-A-S-H gels can be very easily distinguished in 0-3-SCSW and 1-1-SCSW specimens, because in these two specimens remained PS-ash particles are small in number. Conversely, for 0-1-SCSW and 1-3-SCSW specimens, the discrimination between the two categories of matrix gel is not so easy, because there are a relatively large quantity of remained PS-ash particles in these specimens, exhibiting sharp and elongated shapes. More details will be explained again in Section 3.5.

The concentration of aluminum included in the matrix gels is generally very low, as indicated by blue and light blue color codes, corresponding to N-A-S-H and C-A-S-H gels, respectively. There was a reversal concentration of Al between so-called C-A-S-H gels (island pattern) and so-called N-A-S-H gels (sea pattern), indicated by Al-color codes. This result was in contradict with the results of SEM-EDS point analysis, as mentioned in Section 3.5. It is probably due to the influence of highly Al-bearing relicts of host mineral observed in C-A-S-H gels. The low incorporation tendency of Al_2_O_3_ component is consistent with other literature data, that is alkali-free C-A-S-H gels are generated with Al_2_O_3_ in the range of only 6–13 mol %, as plotted in SiO_2_-CaO-Al_2_O_3_ ternary diagram [19]. For the geopolymers prepared from urban refuse incineration ash slags (U-slags) cured at 80 °C, it was found that the Al_2_O_3_ content in GP matrix gels was 10–13 mol % too, when CaO, Al_2_O_3_, and SiO_2_ components were looked over as main compositions of GP matrix composed of N-C-A-S-H, and were plotted in a SiO_2_-CaO-Al_2_O_3_ ternary diagram in dry base [18]. Another study [17] shows in the hybrid cement geopolymers prepared from fly ash and Portland cement mixture cured at 21 °C for one year, the Al_2_O_3_ content is also very low, falling in the range of 0–18 mol %, mainly 1–10 mol %. More details will be mentioned in Section 3.5.

**Figure 3:**
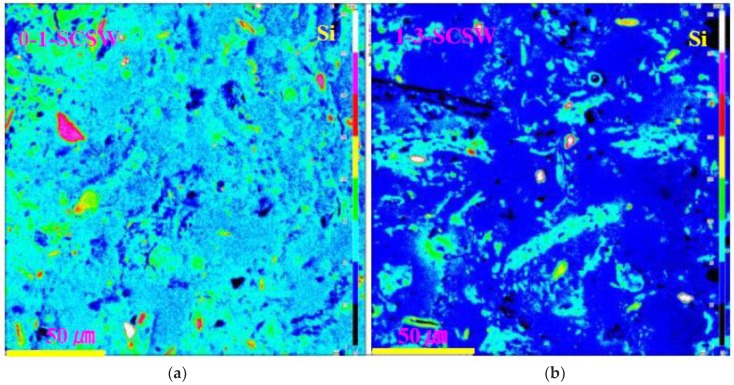
Selected Si-distribution maps of PS-GP taken by EPMA. (**a**) Series 0-1-SCSW; (**b**) Series 1-3-SCSW.

As mentioned above, PS-ash particles decomposed and converted to the GP-minerals other than N-A-S-H and C-A-S-H gels. Among the GP-minerals, quartz remained intact, as seen in the Si-maps (Figure 3), in which the quartz particles are indicated by red and white color codes.

#### 3.4.2. Chlorine Distribution

**Figure 4:**
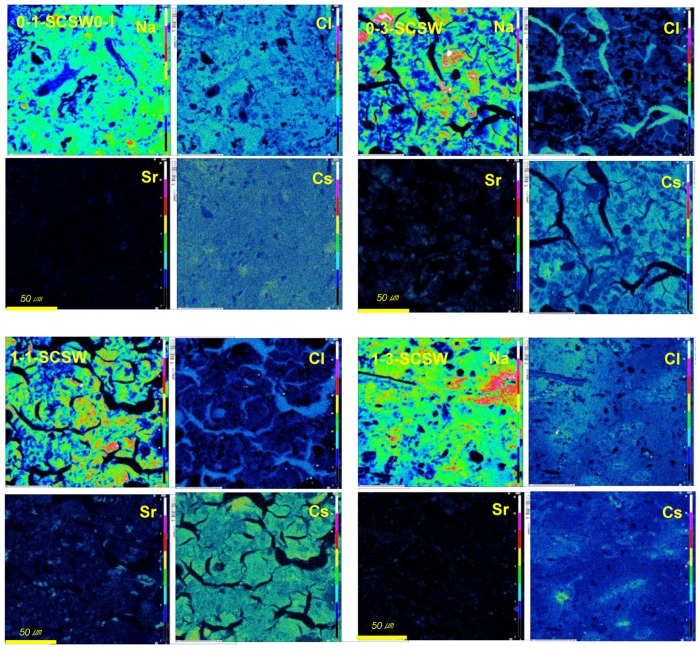
Element distribution comparison of Na with Sr and Cs as well as Cl, taken by EPMA. All the scalar bars are 50 μm in length.

As shown in Figure 4, chlorine is distributed overall, and its distribution strongly correlates with that of sodium. In other words, chlorine has a preference to incorporate into N-A-S-H rather than C-A-S-H gels. This was also confirmed by point analyses using SEM-EDS, as shown in Table 6 and Table 7. These two categories of geopolymer gel constituting the matrices of GP body can also be seen in the Na-maps as well as the Al-maps, as clearly shown by light and shade patterns of color codes. The chlorine preference of N-A-S-H may be due to its zeolite-like structure having sodalite cages as sub-cells which can accommodate much chlorine. According to a study reporting on LCFA (low calcium fly ash)-based GP, a zeolite-like lattice image taken by a high-resolution electron microscope (HREM) shows that there are partial precipitates of zeolite A-like crystals with sodalite cages in the matrix N-A-S-H gel [20]. From this result, it is estimated that N-A-S-H gel may have sodalite cages in its gel structure.

In present study, chlorine was also found in the fissures generated due to the delayed foaming. It seems that the presence of chlorine in the fissures was a result of seeping out from the GP matrix. However, counterpart sodium was scarcely found in the maps. SEM-EDS analysis at certain point shows a pronounced presence of chlorine up to 70–80 mol % in addition to 7–8 mol % SiO_2_. More details are now under investigation and the results will be reported in the near future.

#### 3.4.3. Strontium and Cesium Distribution

**Figure 5:**
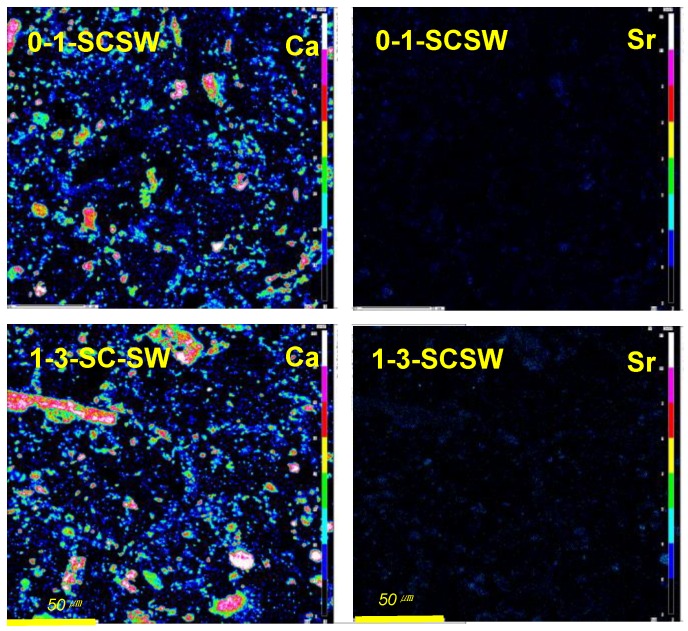
Exemplar of Ca and Sr distribution maps taken by EPMA. Picture field is the same as Figure 4, and all the scalar bars are 50 μm in length.

As shown in Figure 4, strontium is concentrated at several domains, while cesium is entirely scattered though there are some domains. From Figure 4 and Figure 5, it is concluded that the presence of strontium strongly correlates with calcium, while cesium is greatly associated with sodium. Now, we cannot yet conclude certainly the compatibility of elements or compounds, because the dosages of strontium and cesium are smaller. Speculations on the basis of these maps and the literatures will be explained below.

Regarding compatible partnership of strontium, the first candidate is calcite. According to the literatures [21,22], Sr^2+^ can be incorporated into calcite. Thereby, simultaneous incorporation of Mg^2+^ would promote the Sr^2+^ incorporation to compensate the lattice distortion due to the gap of ionic radii of Ca^2+^ as expressed (0.131 + 0.072)/2 = 0.10 nm. The ionic radii are Sr^2+^ (0.131 nm—IX), Mg^2+^ (0.072 nm—VI) and Ca^2+^ (0.10 nm—VI), respectively [23,24], in which the Roman numerals indicate coordination numbers. Therefore, calcite has a high potential to accommodate strontium and other divalent cations. 

The second candidate is calcite-aragonite overgrowth. According to literature [25], strontium plays an important role in the growth of calcite-aragonite alternate layer triggered by seasonal change of hot spring water such as temperature, pH and so on. This fact was found in a hot spring in Japan. Thereby, Sr^2+^ acts as nucleation agent of metastable formation of aragonite overgrowth on calcite. Present PS-ash fillers have sufficient Mg-potentials, as found in Table 1.

Based on the above research data, we consider the reason of the stable immobilization of strontium as follows. The calcite is soluble in acidic solution. However, it might be covered by geopolymer matrix gels so that its dissolution is hindered. Accordingly, the strontium combined in the calcite does not easily dissolute even in acidic environment. We identified the calcite by XRD in the PS-GP specimens, but we have not yet detected the calcite itself as well as the calcite-aragonite overgrowth from the SEM-EDS maps of all the PS-GP specimens. Maybe this is because the calcite was scarce and in slanted distribution in the scanned fields of specimens. Otherwise, the calcite was extremely small in size beyond the resolution power of SEM apparatus.

The third candidate is the C-A-S-H gel that is rich in calcium. Specifically, simultaneous incorporation of magnesium may be important in the case that calcite compensates lattice distortions. Magnesium source is talc and forsterite from the PS-ashes. Actually, relatively high content of MgO has been detected in some C-A-S-H gels, as shown in Table 6 and Table 7.

The fourth candidate is so-called ‘plagioclase gels’, presumably comprising faujasite, (Na_2_CaMg)_3.5_(Al_7_Si_17_O_48_)·32H_2_O, of zeolite family, of which presence is limited solely to 0–1 and 1–3 series samples until one year age, as seen in Figure 1. Therefore, incorporation of strontium into Ca-rich plagioclase gels themselves and/or faujasite-Ca is plausible for the PS-GP specimens besides the candidates mentioned above. The details of plagioclase gels can be referred to Section 3.5.

Discrete formation of strontianite, SrCO_3_, was reported in a literature about metakaolin-based geopolymer studied by HREM [26]. However, this is not directly applicable to present study, because present PS-ash fillers have multi-phases rather than single phase filler. Metakaolin only produces N-A-S-H gels, which have little compatibility to strontium incorporation, as mentioned above. Accordingly, strontium is obliged to appear as strontianite in metakaolin, combined with carbon dioxide. 

Next, we continue to discuss incorporation of cesium. 

About compatible partnership of cesium, the first candidate is N-A-S-H gel, as clearly seen in 0-3-SCSW and 1-1-SCSW specimens without faujasite. Incidentally, incorporation of cesium into matrix gels was elucidated in metakaolin-based and LCFA-based geopolymers, respectively [7,10]. These geopolymers, using single sort of active filler, have one kind of matrix gel.

Besides, faujasite-Na would be the third candidate of cesium accommodation. Cs-faujasite was encountered in XRD identification file, ICDD 01-079-1887, Cs_39.36_Na_40.80_Al_96_Si_96_O_384_·H_2_O_164.48_, so that faujasite is versatile to gather monovalent and divalent cations into its structure. Another versatile mineral to accommodate cesium and strontium is herschelite, which has been renamed chabazite-Na, (Na_2_, K_2_, Ca, Sr, Mg)_2_[Al_2_Si_4_O_12_]_2_·12H_2_O. Chabazite-Na formation has been reported in studies [27,28,29]. However, it was not detected at all from our specimens prepared in this study as well as our previous studies [4,5].

### 3.5. SEM-EDS Analysis

#### 3.5.1. N-A-S-H and C-A-S-H Issue

**Figure 6:**
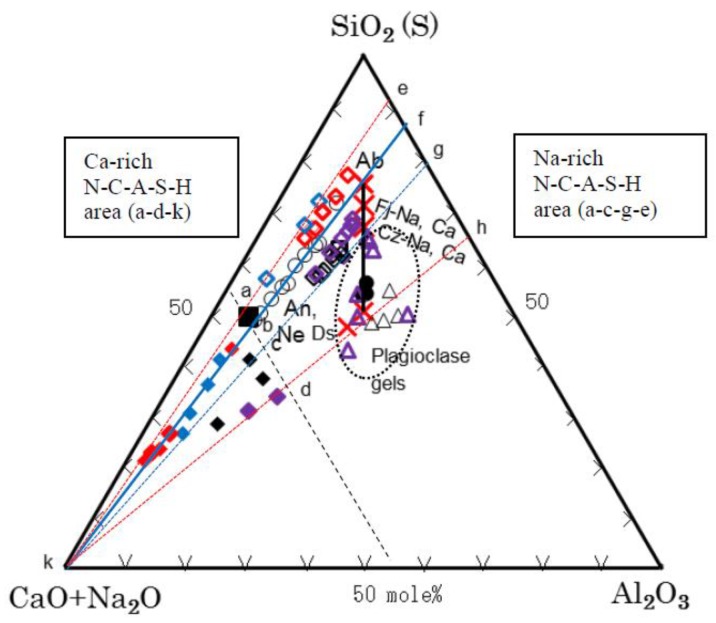
Ternary diagram of so-called N-A-S-H and C-A-S-H matrix gels in terms of SiO_2_-(CaO + Na_2_O)-Al_2_O_3_. ◇-◆-△: 0-1-SCSW, in black. 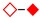: 0-3-SCSW, in red. 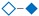: 1-1-SCSW, in blue. 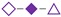: 1-3-SCSW, in purple. ○: Yamaguchi et al., 2013. **□**-■: Yip et al., 2005. ●: Iwahiro et al., 2002. Unfilled marks except triangles are for Na-rich N-C-A-S-H. Filled Marks are for Ca-rich N-C-A-S-H. Unfilled triangles are for plagioclase gels. Cross marks in red indicate positions of Ab: albite; An: anorthite; Ne: nepheline; Ds: davidsmithite; Fj: faujasite; and Cz: chabazite, respectively. Solid blue line: main track of trend line (CN-Ab). Dotted blue line: sub-track of trend line. Red lines: frontiers of plots. Black broken line: estimated boundary between Na-rich N-C-A-S-H (N-A-S-H) and Ca-rich N-C-A-S-H (C-A-S-H).

**Figure 7:**
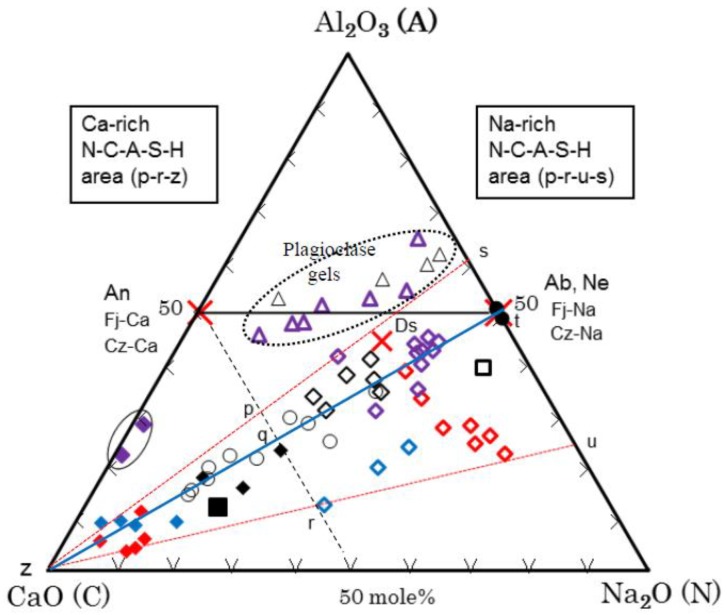
Ternary diagram of so-called N-A-S-H and C-A-S-H matrix gels in terms of Al_2_O_3_-CaO-Na_2_O. The solid oval circle indicates nearly pure C-A-S-H gels. Other notations are the same as Figure 6.

**Figure 8:**
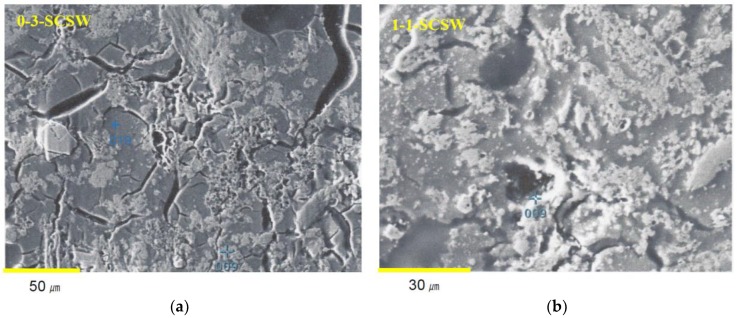
Fine SEM images for showing N-A-S-H and C-A-S-H gels in dark and bright contrast. The numbers of analyzed points correspond with Table 6 and Table 7. (**a**) Series 0-3-SCSW; (**b**) Series 1-1-SCSW.

All the analytical data of components of N-A-S-H and C-A-S-H gels are summarized in Table 6 and Table 7. Figure 6 and Figure 7 show a ternary diagram of SiO_2_-(CaO + Na_2_O)-Al_2_O_3_ and Al_2_O_3_-CaO-Na_2_O, respectively, by molar ratio. As exemplified in Figure 2 that was the results of EPMA, N-A-S-H and C-A-S-H gels can be seen in sea and island patterns. Exemplified SEM images under high magnification power are shown in Figure 8, in which the sea and island patters appear more vividly, and sponge-like texture of C-A-S-H gel, a proof of topotactic precipitation, is also clearly observed.

It is thought that N-A-S-H gels precipitated in the GP-liquors by way of so-called “through solution process”, while C-A-S-H gels were formed by way of so-called ‘topotactic process’ precipitating on the surfaces of active filler particles. It is likely that most of the constituent minerals of the PS-ashes except quartz turned into N-A-S-H and C-A-S-H gels in the 0-3-SCSW and 1-1-SCSW. To the contrary, in the 0-1-SCSW and 1-3-SCSW, a considerable number of PS-ash particles remained even in the one-year-old specimens, maintaining original elongated shapes in back scattered electron images of EPMA. On the other hand, in the SEM images, the sea and island texture patterns can be well clarified. In addition, it should be stressed that the grains exhibited bright in color that is white to light gray so as to look like C-A-S-H gels at a glance on the display screen. However, some of them actually possessed Al-rich N-A-S-H gel-like compositions. This kind of gel is temporarily called ‘plagioclase gels’ here, noted as PL in Table 7**,** and surrounded with a dotted oval circles in Figure 6 and Figure 7. The plagioclase gels appear generally in elongated shape, which may be pseudomorphs after the PS-ash minerals connect most probably with faujasite formation, specifically anorthite included in the PS-ashes [4,5]. However, they do not always appear in elongated shape, but sometimes in round shape.

Moreover, according to the Al_2_O_3_-CaO-Na_2_O diagram of Figure 7, and the XRD results of Figure 1 where faujasite peaks are clear for 0-1-SCSW but are slight for 1-3-SCSW, some of the plagioclase gels maybe converted to crystalline faujasite in form of solid solution consisting of faujasite-Ca and faujasite-Na end-members. There may be some participation of faujasite-Mg, but MgO-component is omitted from the ternary diagrams.

From the SiO_2_-(CaO + Na_2_O)-Al_2_O_3_ diagram shown in Figure 6, alignments alongside CN-Ab join can be seen for the plots of series 0-3-SCSW (red marks) and series 1-1-SCSW (blue marks), where CN denotes CaO + Na_2_O. However, the alignments are somewhat different between 0-1-SCSW (black marks) and 1-3-SCSW (purple marks). Specifically, compositional ratios of their C-A-S-H draw out largely from the CN-Ab join, probably due to the formation of plagioclase gels. The plagioclase gels were only found in 0-1-SCSW and 1-3-SCSW specimens, both of which had faujasite. Hence, part of the plagioclase gels would be crystalline faujasite.

The Al_2_O_3_-CaO-Na_2_O diagram (refer to Figure 7) is explained here. This diagram was prepared to separate the CN into two components that are CaO and Na_2_O, respectively. The plots are concentrated alongside C-Ab join, where C denotes apical CaO. However, large deviations from the C-Ab join were recognized for N-A-S-H gels of 1-1-SCSW (blue marks) and 0-3-SCSW (red marks), of which N-A-S-H gels were plotted toward Na-rich region. The plots of 0-3-SWSC may make a trend line alongside N-Ds join, where N denotes Na_2_O and Ds is davidsmithite, (Ca, □)_2_Na_6_Al_8_Si_8_O_32_, where the box is vacancy. On the other hand, it is noted that C-A-S-H gels of 1-3-SCSW (purple marks) contain very small amounts of N-components so that they can be regarded as nearly genuine C-A-S-H gels.

Lastly, ‘plagioclase gels’ are discussed. Their characteristics were explained in Section 3.4.3 as well as in the middle of this subsection. The plots of these gels locate in the vicinity of Ab-An join. Otherwise, that is also equivalent to (Fj-Na)-(Fj-Ca) join and (Cz-Na)-(Cz-Ca) join in the Al_2_O_3_-CaO-Na_2_O diagram. The trend is somewhat upward and downward against the exact Ab-An join. Presumably, some of these gels partially converted to crystalline faujasite as mentioned, especially in case of 0-1-SCSW. 

#### 3.5.2. Literature Data for Supplement

When using metakaolin (MK) and Na-silicate GP-liquors to prepare GP, it is quite naturally considered that N-A-S-H gels form as binding matrices in geopolymer. Low calcium fly ash (LCFA) is the same. On the other hand, geopolymer prepared from ground granulated blast-furnace slag (GGBS or GGBFS), generally together with LCFA corresponding to ASTM class F, may generate C-A-S-H gels other than N-A-S-H gels. However, the difference between these two extreme gels has been not yet well understood whether solid solutions such as plagioclase exist in the two extreme gels or not. However, there have been so many studies on N-A-S-H and C-A-S-H gels as seen in literatures (e.g., [6,7,18,19,20,26,27,28,29,30,31,32,33]).

On the other hand, according to Yamaguchi et al. [18], urban waste incineration ash slag (U-slags) has a wide range of compositions, depending on incineration plants. Their compositions are plotted alongside the first hydraulic line in the SiO_2_-CaO-Al_2_O_3_ ternary diagram. They prepared monolithic geopolymer materials by using the U-slag powder activated with Na-silicate GP-liquor corresponding to the GP-liquor #0 used in present study and curing at 80 °C. They also made point-analyses of matrix compositions by SEM-EDS, using polished specimens. Their results provided useful information on the N-A-S-H and C-A-S-H issue. Thus, we rearranged their data in this study and the results obtained are also shown in Figure 6 and Figure 7**.**

The principal components of SiO_2_, CaO, Al_2_O_3_, and Na_2_O were considered here. These oxide components account for 85~95% of the total compositions. The 10 points based on 10 analytical data from 5 different specimens are plotted in Figure 6 and Figure 7**,** together with the data of this study. The data in the literature [18] strongly indicate that the N-A-S-H gels comprise the CaO-component in which the plots run from the apical CN point toward Ab point. In other words, the N-A-S-H gel of Ab composition in dry base changes to be N-C-A-S-H gel as a result of CaO incorporation. Furthermore, this terminal Ab point was actually confirmed, when possessing N/A molar ratio 1.0 in the attached Al_2_O_3_-CaO-Na_2_O diagram, in which extending trend line from apical CN point to Ab point is clearly noted for reflecting the substitution of Na with Ca. However, independent presence of C-A-S-H gels was not encountered at all in their SEM images (unpublished). Since only U-slag was used as single-phase active filler in the preparation of monolithic geopolymer materials, it is quite natural to consider that only one kind of matrix gel was yielded. Otherwise, it is probable that C-A-S-H components were incorporated into N-A-S-H gels due to high temperature curing at 80 °C.

In addition, genuine N-A-S-H gels synthesized from fluidal reagents are not composed of albite composition but composed of nepheline-rich compositions, as plotted along nepheline (Ne)-albite (Ab) join [30]. The data in the literature were picked up for the gels synthesized only under the alkaline conditions over pH 12. When plotted along the kalsilite–orthoclase join, the same tendency was able to find for the K-analog of K-A-S-H [31]. Accordingly, there is a discrepancy between genuine synthetic gels and in-situ matrix gels presumably due to the difference in kinetics of solution and suspension of source materials. Incidentally, albite was detected as main phase in GGBS-LCFA based geopolymer pastes heated at 1150 °C [32] so that the matrices have a high albite potential in their compositions in this case.

Other literature data obtained from MK-GGBFS based geopolymer matrices cured at 40 °C [33] are recalculated into oxide components and also plotted in Figure 6 and Figure 7, in which original oxygen data were neglected, since energy dispersive spectroscopy (EDS) is incompetent for quantitative analysis of oxygen [34]. Copresence of the two discrete gels, designated as phase A and phase B in the literature, would be described as follows: Phase A is Na-rich composition, N_20.04_-C_3.01_-A_14.81_-S_62.14_-(H) as dry base that may be MK-origin, and Phase B is Ca-rich, N_11.33_-C_33.66_-A_6.16_-S_48.85_-(H) as dry base that may be GGBFS-origin. Accordingly, they are plotted separately from each other. 

Therefore, conventionally named N-A-S-H and C-A-S-H gels should be called Na-rich N-C-A-S-H and Ca-rich N-C-A-S-H gels. The formation areas of N-A-S-H gels encompass a-c-g-e and p-r-u-s trapezoids, and those of C-A-S-H gels are a-d-k and p-r-z triangles in the SiO_2_-(CaO + Na_2_O)-Al_2_O_3_ and the Al_2_O_3_-CaO-Na_2_O ternary diagrams in Figure 6 and Figure 7, respectively. It is noted that, when viewing from the apical CN point, no N-A-S-H gels were found beyond Ab-An join. The boundary between the two extreme gel phases is estimated to be S_55_CN_45_-CN_45_A_55_ line and A_50_C_50_-C_50_N_50_ line, when GP is cured at low temperature up to 40 °C. However, at high temperature probably high above 40 °C, both gels would be incorporated to yield a single-phase gel.

Although similar results as in Figure 6 were also obtained for N-A-S-H and C-A-S-H gels of one year old hybrid cement body, as shown in the literature [17], the big difference from the present study is whether to consider the Na_2_O component or not. In their study, geopolymers of the hybrid cement aged four weeks showed separate distribution between N-A-S-H and C-A-S-H gels and eventually they lined up after one year and the distribution gap between them disappeared, which is very similar to the results described in [18]. This is an issue of non-equilibrium and equilibrium or unstable and stable argument of the two extreme gels with the elapsed time. Our results on PS-GP showed a small gap between N-A-S-H and C-A-S-H gels for one year old samples and were different from the results of the one year old hybrid cement body which showed no gap. Further investigation is required to solve this problem.

## 4. Conclusions

In this study, a novel technology was developed for treating hazardous water contaminated with radionuclides by geopolymer technique. The geopolymers (GP) were prepared by two kinds of PS-ash as active fillers and two kinds of alkaline activator called GP-liquor adding seawater. Non-radioactive Sr-nitride and Cs-nitride were added as surrogates as much as 1% by mass, respectively, of the PS-ashes. All the geopolymers were cured in the ambient air. The immobilization ratios of strontium and cesium were measured at several ages up to one year, and immobilization mechanism as well as PS-ash based geopolymer’s compositions and textures were investigated in detail. The main results obtained are summarized as follows:

When using the GP liquors without seawater, immobilization ratios of strontium and cesium were unstable, fluctuating with material age. However, when the seawater was mixed into the GP-liquors, this deficiency was extremely improved. Specifically, the inexpensive GP-liquor #1, which did not contain caustic soda, effectively stabilized the immobilization ratios, compared to the GP-liquor #0 with NaOH. 

When mixing seawater into the GP-liquors, the liquor/filler ratio (L/F) of PS-ash based geopolymer (PS-GP) could be set larger, compared to the PS-GP without seawater-mixing. Higher L/F, however, yielded a lower strength of PS-GP. Since the PS-ashes contain free metallic aluminum, hydrogen was generated during the solidification of PS-GP. Delayed foaming after setting was observed in some PS-GP.

Strontium distribution is closely related to calcium, whereas cesium is widespread. Strontium may be accommodated into a calcite, calcite-aragonite complex, Ca-rich N-C-A-S-H gels, conventionally so-called C-A-S-H, and plagioclase gels—faujasite-Ca complex. Magnesium, originated from talc and forsterite, may promote the incorporation of strontium with these minerals. On the other hand, cesium may be incorporated into Na-rich N-C-A-S-H gels, conventionally so-called N-A-S-H, and plagioclase gels—faujasite-Na complex.

Based on the SEM-EDS analysis, generally so-called N-A-S-H and C-A-S-H gels should be called Na-rich N-C-A-S-H and Ca-rich N-C-A-S-H gels, respectively. When plotted, the analytical data in the ternary diagrams of SiO_2_-(CaO + Na_2_O)-Al_2_O_3_ and Al_2_O_3_-CaO-Na_2_O, most of the two gels concentrate alongside the CN-Ab join, which can be regarded as trunk trend line. However, some deviations from this line are noted for geopolymer specimens possessing “plagioclase gels”, which comprise crystalline faujasite to yield plagioclase gels-faujasite complex. The faujasite is formed at ambient temperature, and its most probable source mineral is anorthite. 

## 5. Patents

The authors have already applied for a patent in Japan for the technology described in this paper for stabilizing of immobilization of strontium and cesium in PS-GP.

## Figures and Tables

**Table 1:** Specifications of GP-liquor used.

Aqueous Solution	Specific Gravity	Salt Concentration (%)	
	(S.G.)	Bulk	Chlorine
Seawater	1.04	3.445 ^1^	1.898 ^1^
JIS No. 1 stock solution	1.54	-	-
GP-liquor #0SW ^2^	1.30	1.357	0.748
GP-liquor #1SW ^2^	1.27	1.389	0.765

^1^ Standard concentration of Pacific Ocean, ^2^ Seawater mixing.

**Table 2:** Chemical compositions determined by XRF and physical constants of air-dried PS-ash [4,5].

PS-ash	SiO_2_	TiO_2_	Al_2_O_3_	Fe_2_O_3_	MnO	CaO	MgO	Na_2_O	K_2_O	P_2_O_5_	SO_3_	Cl	Others ^1^
OTo3	30.94	1.55	37.37	1.88	0.03	18.57	3.58	0.33	0.81	1.56	2.71	0.41	0.28
N45	21.87	0.77	13.75	2.58	0.37	34.95	10.44	0.80	0.78	3.56	9.25	0.42	0.45
**PS-ash**	**Key**	**Apparent Density, g/cm^3^**				**Specific Surface Area, cm^2^/g, Blaine**					**Total of XRF Analysis**		
OTo3	1	2.50				6460					100.02 %		
N45	3	2.26				5680					99.99 %		

**^1^** Including ZnO, CuO, BaO, SrO, NiO, PbO, ZrO_2_, CeO_2_, Cr_2_O_3_, Bi_2_O_3_, etc. SrO is 0.023 and 0.066% for OTo3 and N45, respectively. Cs_2_O is not detected at all. Loss on ignition (LOI) heat-treated at 1000 °C for 2 h is 6.00 (1.66)% and 22.10 (12.49)% for OTo3 and N45, respectively, where in parentheses is indicated H_2_O (-) dried at 105 °C for 2 h. LOI and H_2_O (-) are excluded from the total of XRF analysis.

**Table 3:** Results on bulk density and flexural strength of PS-GP.

Hardened Body	Seawater Mixed	Active Filler	L/F ^1^	Flexural Strength (MPa)	Bulk Density (g/cm^3^)				
					Age (Week)				
Series	GP liquor	-	-	4 weeks	4	6 (4 + 2) ^2^	12	24 ^5^	52 ^5^ (1 year)
(a) PS-GP using seawater-mixed GP-liquors									
0-1-SCSW ^3^	#0SW	OTo3	1.94	0.68	1.09	0.71	0.67	0.70	0.65
0-3-SCSW	”	N45	1.50	0.81	1.48	1.10	0.95	1.07	1.02
1-1-SCSW ^4^	#1SW	OTo3	1.50	1.69	1.56	1.04	1.09	1.06	1.02
1-3-SCSW	”	N45	1.50	0.92	1.53	1.02	0.98	1.01	0.96
(b) PS-GP using non-seawater-mixed GP-liquors									
0-1-SC ^3^	#0	OTo3	1.50	1.22	1.29	0.84	0.82	-	-
0-3-SC	“	N45	1.20	0.99	1.49	1.08	0.97	-	-
1-1-SC ^4^	#1	OTo3	1.50	1.07	1.58	1.05	1.02	-	-
1-3-SC	“	N45	1.20	1.19	1.54	1.05	0.98	-	-

**^1^** Liquor/Filler ratio by mass; ^2^ Refer to the text; ^3^ Marked foaming; ^4^ Slight foaming; ^5^ In case of PS-GP using non-seawater-mixed GP-liquors, no marked change of bulk density was observed after 12 weeks so that the same data at 12 weeks were applied for 24 and 52 weeks.

**Table 4:** Exemplified data for calculating immobilization ratios of Sr^2+^ and Cs^+^ in PS-GP.

Age	% Filler	12.5 g Sample	Surrogates (mg)			ICP, 421 nm	ICP, 459 nm
52 Weeks		(g)	As Nitrate	Sr^2+^	Cs^+^	Sr^2+^ (ppb)	Cs^+^ (ppb)
(a) Seawater-mixed PS-GP							
Liquor #0SW							
0-1-SCSW	57.04	7.13	71.3	29.5	48.6	610	23,410
0-3-SCSW	58.04	7.26	72.6	30.1	49.5	1770	19,900
Liquor #1SW							
1-1-SCSW	61.18	7.65	76.5	31.7	52.2	3340	0
1-3-SCSW	63.75	7.97	79.7	33.0	54.4	7840	0
	125 g Leaching Solution			Dissolution Ratio		Immobilization Ratio	
	Sr^2+^ (µg)	Cs^+^(µg)		Sr^2+^ (%)	Cs^+^ (%)	Sr^2+^ (%)	Cs^+^ (%)
Liquor #0SW							
0-1-SCSW	76.25	2926.25		0.26	6.02	99.74	93.98
0-3-SCSW	221.25	2487.50		0.74	5.02	99.26	94.98
Liquor #1SW							
1-1-SCSW	417.50	0		1.32	0	98.68	100
1-3-SCSW	980.00	0		2.97	0	97.03	100
(b) Non-seawater-mixed PS-GP							
Liquor #0							
0-1-SC	62.93	7.87	78.7	32.6	53.7	510	0
0-3-SC	61.44	7.68	76.8	31.8	52.4	380	0
Liquor #1							
1-1-SC	70.41	8.80	88.0	36.4	60.0	4420	26,680
1-3-SC	71.43	8.93	89.3	37.0	60.9	O.S.^1^	22,060
	125 g Leaching Solution			Dissolution Ratio		Immobilization Ratio	
	Sr^2+^ (µg)	Cs^+^ (µg)		Sr^2+^ (%)		Sr^2+^ (µg)	Cs^+^ (µg)
Liquor #0							
0-1-SC	63.75	0		0.20	0	99.80	100
0-3-SC	47.50	0		0.15	0	99.85	100
Liquor #1							
1-1-SC	552.50	3335.00		1.52	5.56	98.48	94.44
1-3-SC	O.S.^1^	2757.50		O.S.^1^	4.53	O.S.^1^	95.47

**^1^** Over-scale (O.S.) took place due to too much concentrations of testing leachate to measure and no more measurements were conducted by further dilutions. Table 5 is the same.

**Table 5:** Dissolution test results of PS-GP.

Hardened Body	Seawater Mixed	Active Filler (Key)	Immobilization Ratio (%) for Each Age (Week)							
Series	GP-Liquor		6 (4 + 2)		12		24		52 (1 Year)	
			Sr^2+^	Cs^+^	Sr^2+^	Cs^+^	Sr^2+^	Cs^+^	Sr^2+^	Cs^+^
(a) PS-GP using seawater mixed GP-liquors										
0-1-SCSW	# 0SW	OTo3 (1)	99.72	96.99	99.59	100	99.70	100	99.74	93.98
0-3-SCSW	“	N45 (3)	99.61	93.06	99.72	100	99.41	100	99.26	94.98
1-1-SCSW	# 1SW	OTo3 (1)	99.30	93.08	97.61	100	98.84	100	98.68	100
1-3-SCSW	“	N45 (3)	97.70	95.22	95.34	100	97.30	100	97.03	100
(b) PS-GP using non-seawater-mixed GP-liquors										
0-1-SC	# 0	OTo3 (1)	99.79	97.24	99.80	O.S.	99.89	97.48	99.80	100
0-3-SC	“	N45 (3)	99.83	71.54	99.83	O.S.	99.88	98.30	99.85	100
1-1-SC	# 1	OTo3 (1)	98.23	96.58	98.27	90.95	98.59	98.77	98.48	94.44
1-3-SC	“	N45 (3)	O.S.	71.06	91.73	46.16	O.S.	95.65	O.S.	95.47

**Table 6:** Results of SEM-EDS point-analysis for seawater-mixed PS-GP using NaOH-containing GP-liquor #0SW at 52 weeks.

**Phase**		**Specimen 0-1-SCSW**											
EDS	Screen	Point	SiO_2_	TiO_2_	Al_2_O_3_	Fe_2_O_3_	CaO	MgO	Na_2_O	K_2_O	P_2_O_5_	SO_3_	Cl
	C-A-S-H ^1^	S7	25.76	0.61	10.62	0.34	39.81	3.87	16.29	0.17	1.11	0.38	1.06
	C-A-S-H	S9	38.06	0.34	10.00	0.26	36.61	2.61	9.27	0.85	0.91	0.25	0.83
	N-A-S-H ^2^	S11	55.47	0.17	13.95	0.29	11.62	1.52	11.47	0.62	0.35	0.47	4.09
	N-A-S-H	S12	56.21	0.22	14.72	0.29	9.32	1.34	12.00	0.76	0.30	0.58	4.27
	N-A-S-H	S13	54.48	2.16	12.83	0.39	9.53	2.12	12.53	0.65	0.40	0.65	4.27
	N-A-S-H	S15	52.51	0.28	13.31	0.38	15.53	2.32	10.93	0.58	0.36	0.45	3.34
	N-A-S-H	S16	51.56	0.16	12.18	0.40	15.14	1.71	12.28	0.58	0.61	0.62	4.74
	N-A-S-H	S17	52.67	0.38	13.30	0.47	10.51	1.33	14.81	0.63	0.61	0.54	4.75
	C-A-S-H	S18	31.91	2.40	12.80	0.61	27.41	4.39	14.83	0.31	2.89	0.83	1.61
PL ^3^	C-A-S-H	S19	50.02	0.20	25.21	0.28	3.04	4.10	14.25	0.38	0.59	0.23	1.72
PL	C-A-S-H	S20	44.42	0.49	27.03	0.26	7.76	3.48	13.22	0.33	1.08	0.31	1.62
PL	C-A-S-H	S21	47.23	0.17	30.09	0.18	2.02	1.08	16.96	0.28	0.47	0.27	1.25
PL	C-A-S-H	S22	43.04	1.46	24.79	0.87	16.72	4.26	5.59	0.09	0.66	1.82	0.71
**Phase**		**Specimen 0-3-SCSW**											
EDS	Screen	Point	SiO_2_	TiO_2_	Al_2_O_3_	Fe_2_O_3_	CaO	MgO	Na_2_O	K_2_O	P_2_O_5_	SO_3_	Cl
	N-A-S-H	S7	60.13	0.14	7.54	0.25	4.23	2.19	21.77	0.90	0.15	0.27	2.42
	N-A-S-H	S8	61.42	0.11	8.16	0.21	4.18	2.66	19.03	0.67	0.14	0.38	3.03
	N-A-S-H	S9	64.49	0.11	7.95	0.15	4.45	1.99	16.12	0.96	0.17	0.46	3.15
	N-A-S-H	S10	61.75	0.13	7.66	0.34	5.19	2.85	18.34	0.87	0.26	0.22	2.40
	C-A-S-H	N9	24.07	0.41	4.01	0.12	55.35	5.38	8.85	0.18	0.75	0.43	0.45
	C-A-S-H	N10	21.06	0.00	3.04	0.25	61.58	3.71	8.96	0.12	0.52	0.40	0.36
	C-A-S-H	N13	18.72	0.21	3.49	1.08	55.43	11.93	3.55	0.18	3.80	0.88	0.74
	C-A-S-H	N14	19.59	0.10	2.65	0.09	64.29	3.79	8.26	0.14	0.34	0.35	0.39
	C-A-S-H	N15	35.46	0.17	5.43	0.64	37.81	13.77	4.60	0.21	0.65	0.33	0.93
	N-A-S-H	N17	64.04	0.05	7.83	0.36	5.75	2.46	14.76	0.67	0.22	0.31	3.55
	N-A-S-H	N19	66.32	0.11	8.40	0.24	5.33	3.89	11.44	0.58	0.23	0.39	3.07
	N-A-S-H	N20	70.21	0.10	8.29	0.28	4.56	3.61	8.63	0.72	0.29	0.27	3.03

**^1^**^,2^ Conventionally accepted nomenclatures are used in this table. Actually, they are Na-rich N-C-A-S-H for N-A-S-H and Ca-rich N-C-A-S-H for C-A-S-H, respectively. ^3^ Plagioclase gels.

**Table 7:** Results of SEM-EDS point-analysis for seawater-mixed PS-GP using non-NaOH-containing GP-liquor #1SW at 52 weeks.

**Phase**		**Specimen 1-1-SCSW**											
EDS	Screen	Point	SiO_2_	TiO_2_	Al_2_O_3_	Fe_2_O_3_	CaO	MgO	Na_2_O	K_2_O	P_2_O_5_	SO_3_	Cl
	N-A-S-H	S1	65.33	0.03	6.26	0.12	7.35	2.04	12.62	0.89	0.18	0.35	4.82
	N-A-S-H	S2	50.17	0.38	4.92	0.41	18.55	4.99	15.36	0.54	0.45	0.51	3.72
	N-A-S-H	S3	59.35	0.00	5.88	0.46	10.34	2.95	13.26	0.62	0.26	0.49	6.39
	C-A-S-H	S4	22.28	0.32	5.73	0.81	55.29	10.11	2.66	0.16	1.18	0.65	0.82
	C-A-S-H	S5	35.14	0.06	4.81	0.40	38.31	10.05	8.57	0.21	0.92	0.67	0.86
	C-A-S-H	S7	29.58	0.15	5.13	0.49	44.67	13.19	3.80	0.18	0.69	0.77	1.34
	C-A-S-H	S9	26.15	0.22	5.27	0.12	50.04	9.85	6.17	0.12	0.64	0.77	0.64
**Phase**		**Specimen 1-3-SCSW**											
EDS	Screen	Point	SiO_2_	TiO_2_	Al_2_O_3_	Fe_2_O_3_	CaO	MgO	Na_2_O	K_2_O	P_2_O_5_	SO_3_	Cl
	N-A-S-H ^2^	S1	56.01	0.61	15.08	0.42	11.34	2.40	9.98	0.56	0.51	1.27	1.81
	N-A-S-H	S2	61.91	0.09	13.53	0.33	5.27	2.36	12.12	0.30	0.66	1.65	1.78
	N-A-S-H	S3	60.49	0.16	13.56	0.22	5.63	2.49	12.99	0.37	0.66	1.36	2.09
	N-A-S-H	S8	61.60	0.11	14.04	0.23	4.33	3.02	12.67	0.27	0.52	1.54	1.65
	N-A-S-H	S9	61.09	0.04	13.52	0.23	5.20	2.56	13.10	0.41	0.41	1.18	2.25
PL ^3^	C-A-S-H ^1^	S4	58.26	0.22	17.41	0.63	6.64	3.18	8.98	1.87	0.44	1.48	0.89
PL	C-A-S-H	S7	45.89	0.46	22.98	0.23	17.04	2.83	8.03	0.24	0.77	1.27	0.25
PL	C-A-S-H	S11	60.37	0.83	17.04	0.27	9.61	3.08	6.58	0.47	0.42	1.06	0.26
PL	C-A-S-H	S12	48.41	0.37	20.42	3.15	14.30	3.42	7.86	0.39	0.64	0.66	0.36
PL	C-A-S-H	S13	56.32	2.85	18.79	0.27	4.60	3.45	11.30	0.58	0.43	0.78	0.62
PL	C-A-S-H	N1	38.39	1.09	23.80	0.47	22.08	5.15	6.37	0.15	1.07	1.12	0.33
PL	C-A-S-H	N2	46.66	0.29	30.87	0.26	2.99	1.86	14.05	0.37	0.49	1.54	0.63
	C-A-S-H	N3	30.11	1.26	16.98	0.42	42.38	4.61	0.93	0.02	1.34	1.14	0.80
	C-A-S-H	N4	28.57	1.47	14.48	0.27	50.18	3.94	0.66	0	0.15	0.18	0.08
	N-A-S-H	N7	52.79	0.08	12.15	0.25	11.84	2.76	15.37	0.25	0.63	1.66	2.23
	N-A-S-H	N9	64.04	0.19	13.39	0.14	3.88	1.43	12.93	0.40	0.22	1.13	2.25
	N-A-S-H	N10	57.02	0.11	12.53	0.26	7.46	2.14	15.76	0.36	0.38	1.67	2.31
	N-A-S-H	N11	59.17	0.18	13.16	0.30	5.91	2.15	13.96	0.32	0.59	1.97	2.30

**^1^**^,2,3^ are the same as Table 6.
